# An Innovative Clustering Hierarchical Protocol for Data Collection from Remote Wireless Sensor Networks Based Internet of Things Applications

**DOI:** 10.3390/s23125728

**Published:** 2023-06-19

**Authors:** Syed Luqman Shah, Ziaul Haq Abbas, Ghulam Abbas, Fazal Muhammad, Aseel Hussien, Thar Baker

**Affiliations:** 1 Telecommunication and Networking (TeleCoN) Research Center, GIK Institute of Engineering Sciences and Technology, Topi 23640, Pakistan; gee2170@giki.edu.pk; 2 Faculty of Electrical Engineering, GIK Institute of Engineering Sciences and Technology, Topi 23640, Pakistan; ziaul.h.abbas@giki.edu.pk; 3 Faculty of Computer Science and Engineering, GIK Institute of Engineering Sciences and Technology, Topi 23640, Pakistan; abbasg@giki.edu.pk; 4 Department of Electrical Engineering, University of Engineering and Technology, Mardan 23200, Pakistan; fazal.muhammad@uetmardan.edu.pk; 5 College of Engineering, University of Sharjah, Sharjah 27272, United Arab Emirates; 6 School of Architecture, Technology and Engineering, University of Brighton, Brighton BN2 4GJ, UK; t.shamsa@brighton.ac.uk

**Keywords:** unmanned aerial vehicles (UAVs), routing protocol, Internet of Things (IoT), wireless sensor networks (WSNs), energy efficiency, network lifetime

## Abstract

Recently, unmanned aerial vehicles (UAVs) have emerged as a viable solution for data collection from remote Internet of Things (IoT) applications. However, the successful implementation in this regard necessitates the development of a reliable and energy-efficient routing protocol. This paper proposes a reliable and an energy-efficient UAV-assisted clustering hierarchical (EEUCH) protocol designed for remote wireless sensor networks (WSNs) based IoT applications. The proposed EEUCH routing protocol facilitates UAVs to collect data from ground sensor nodes (SNs) that are equipped with wake-up radios (WuRs) and deployed remotely from the base station (BS) in the field of interest (FoI). During each round of the EEUCH protocol, the UAVs arrive at the predefined hovering positions at the FoI, perform clear channel assignment, and broadcast wake-up calls (WuCs) to the SNs. Upon receiving the WuCs by the SNs’ wake-up receivers, the SNs perform carrier sense multiple access/collision avoidance before sending joining requests to ensure reliability and cluster-memberships with the particular UAV whose WuC is received. The cluster-member SNs turn on their main radios (MRs) for data packet transmission. The UAV assigns time division multiple access (TDMA) slots to each of its cluster-member SNs whose joining request is received. Each SN must send the data packets in its assigned TDMA slot. When data packets are successfully received by the UAV, it sends acknowledgments to the SNs, after which the SNs turn off their MRs, completing a single round of the protocol. The proposed EEUCH routing protocol with WuR eliminates the issue of cluster overlapping, improves the overall performance, and increases network stability time by a factor of 8.7. It also improves energy efficiency by a factor of 12.55, resulting in a longer network lifespan compared to Low Energy Adaptive Clustering Hierarchy (LEACH) protocol. Moreover, EEUCH collects 5.05 times more data from the FoI than LEACH. These results are based on simulations in which the EEUCH protocol outperformed the existing six benchmark routing protocols proposed for homogeneous, two-tier, and three-tier heterogeneous WSNs.

## 1. Introduction

Wireless sensor networks (WSNs) have become a crucial technology in the proliferation of Internet of Things (IoT) use cases and application scenarios across a wide range of fields. These WSN-based IoT applications include smart industrial monitoring [1], smart transportation [2], real-time tracking and mapping [3], smart agricultural irrigation [4], smart grids [5], smart homes and smart buildings [6], smart cities [2,7], smart exploration [8], and military reconnaissance [9], as well as applications related to safety, stability maintenance, surveying, medical, and health [10]. WSNs are considered a key enabling technology for IoT due to their numerous applications.

A WSN can be described as a large number of small size, low-cost, battery-operated sensor nodes (SNs) that work collaboratively in the field of interest (FoI) to monitor environmental and physical conditions, such as temperature, noise, smog, humidity, pressure, chemical level, light, fire detection, etc. [3,9,11]. Each SN in a WSN is able to sense, process, and transmit data to other SNs or a base station (BS) either directly or upon request. SNs are typically battery-limited and deployed in remote, inaccessible, hazardous, and dynamic environments [3,11,12], making it practically impossible to replace or recharge their batteries. Various factors such as the inaccessibility to the FoI, limited energy resources, and high cost of maintenance contribute to this challenge. Consequently, energy-efficient and effective routing protocols and techniques are necessary to prolong the lifetime of SNs without requiring battery replacement or recharging.

**Figure 1:**
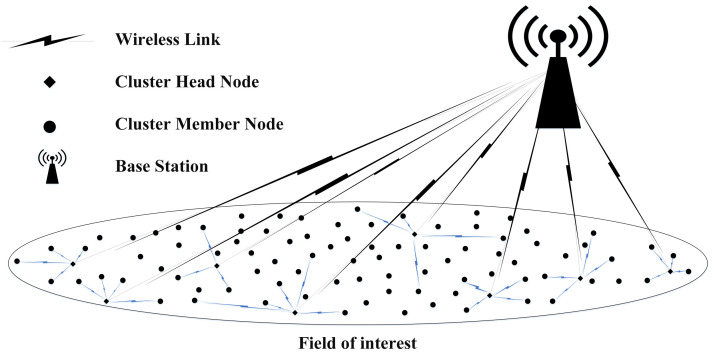
A typical WSN depicting a cluster-based hierarchical routing protocol.

Routing protocols for WSNs are broadly classified into two types: (i) flat and (ii) hierarchical routing protocols [13,14]. In flat routing protocols, all the SNs in WSN play the same role. The packets flow in multihop manner, i.e., the packet is sent to destination or BS through multiple SNs (hops) which are in close proximity to each other; thus, the transmission distance is minimized between the particular SN and the BS, and the energy is saved. Some of the flat routing protocols which are proposed for WSNs are rumor routing (RR) [15], wireless routing protocol (WRP) [16], flooding [17], etc. In flat routing protocols, an SN forwards its data packets to the next SN in the path to the BS. As a result, SNs near to the BS receive and forward data packets repeatedly from multiple SNs due to their physical locations, and they consume more radio energy than the other SNs in the FoI. Thus, these frequently used SNs die early and the problem known as ‘SN hotspot’ occurs in flat routing protocols. To solve this problem, hierarchical routing protocols were proposed. In hierarchical routing protocols, all deployed SNs are divided into groups called clusters, and each cluster selects one of its SNs to serve as the cluster-head (CH), which collects data packets from all its cluster members (CMs). The CMs only sense the surrounding and share the sensed data with the particular CH. The CH consumes more energy and dies early as compared to CMs, because of the reception of data packets from all its cluster member SNs, data aggregation, and forwarding all the collected data packets to the BS. Hence, efficient CH selection from SNs in WSN is a challenging task in hierarchical routing protocols. Such protocols have some benefits over the flat routing protocols, including scalability, load balancing, reducing routing delays, and energy-efficiency. Eminent cluster-based protocols include Power-Efficient Gathering in Sensor Information Systems (PEGASIS) [18], Low Energy Adoptive Clustering Hierarchy (LEACH) [19], Low Energy Adaptive Clustering Hierarchy Centralized (LEACH-C) [20], Stable Energy Protocol (SEP) [21], Threshold sensitive energy Effective Sensor Network protocol (TEEN) [22], Improved Energy Efficient LEACH (IEE-LEACH) [23], and Enhanced Energy Efficient Clustering Approach for Three-tier Heterogeneous Wireless Sensor Networks (EEECA-THWSN) [24]. A typical diagram for clustering-hierarchical routing protocols is shown in Figure 1. However, the most important challenge for these routing protocols is energy conservation.

Moreover, the existing cluster-based routing protocols focus on efficient CH selection in homogeneous WSNs where all the deployed SNs have the same initial energy level [19,20,22,23]. These protocols have also been extended to two-tier WSNs [21] and three-tier heterogeneous WSNs [24], where two and three different types of SNs with distinct initial energy levels are deployed, respectively. However, these protocols assume that the BS is located within the FoI. Practically, the FoI may lie away from the BS, leading to significant challenges. Using existing routing protocols in such cases can result in an unstable WSN, as the CHs may experience early battery depletion (i.e., early death of CH) due to the longer transmission distances. This can lead to reduced data gathering efficiency and impact network performance.

In such scenarios, unmanned aerial vehicle (UAV)-based networks have recently received the attention of the research community due their numerous features such as fast and easy deployment, high-chance of line of sight (LoS) link establishment, reliable connectivity, higher mobility, scalability, and flexibility. In addition, UAVs can survey and scan areas that are difficult to reach by human beings (e.g., oceans and mountains) [25]. Furthermore, due to the adjustable altitude and mobile nature of UAVs, they can move towards the ground users to minimize the transmission distance, and establish a reliable link with low transmit power requirement [26,27,28,29]. Although UAVs as aerial base stations (ABSs) improve the performance of such IoT sensor networks/WSNs significantly, especially in human inaccessible regions such as mountains, oceans, and rural areas, they perform better in collection of data from ground SNs in an energy-efficient way. However, there is a crucial requirement to design an energy-efficient and reliable routing protocol for data collection and delivery through UAVs from remotely deployed SNs.

### 1.1. Novelty and Contributions

In this paper, we propose an Energy-Efficient UAV-assisted Clustering Hierarchical (EEUCH) routing protocol which ensures reliable data collection from ground SNs with the assistance of UAVs. Furthermore, we consider wake-up radio (WuR)-enabled SNs to minimize the energy consumption by diminishing idle listening and overhearing, when there is no UAV for data collection and when multiple wake-up calls (WuCs) are receiving from multiple UAVs, respectively. WuR-enabled SNs are equipped with two radios: (i) main radio (MR) and (ii) wake-up receiver (WuRx). The MR consumes 1000 times more energy, due to reliable data packets transmission, than that of the WuRx [30], which listens to the channel continuously for incoming WuC [31,32]. Thus, WuR works in an on-demand manner [33]. In the proposed model, all the SNs are in sleep mode, in which they sense the surroundings and wait for the WuC from the UAV. As the WuC is detected by the SN’s WuRx, the SN turns on the MR for data packets transmission to the UAV, following the proposed data flow model. When the data packets are received by the UAV, it sends an acknowledgment (AcK) to the SN. The SN receives the AcK, turns off the MR, and goes back into sleep mode. The major contributions of this paper are listed as follows.

- We design and develop a novel cluster-based routing protocol, EEUCH, for WSN-based applications of IoT in which there is no CH election phase. UAVs act as CHs by default and are responsible for the collection of data packets from the FoI.
- We create a novel data flow model in which UAVs act as ABSs. This model uses a combination of clear channel assignment (CCA), carrier sense multiple access/collision avoidance (CSMA-CA), and time division multiple access (TDMA) slots to ensure energy-efficient and reliable data collection.
- We extend the concept of WuR to the network layer in EEUCH for two main reasons: to enhance the network lifetime, stability time, energy efficiency, and the overall data collection from the FoI, and to address the challenge of cluster overlapping.
- A novel UAV-assisted clustering hierarchical model for remote data collection that ensures nonoverlapping and independent clusters and offers a high degree of flexibility in network size is presented.
- The results of our proposed protocol outperform the benchmark cluster-based routing protocols (i.e., homogeneous, two-tier, and three-tier heterogeneous) with significant gains in nine key network performance parameters.

### 1.2. Paper Organization

The rest of the paper is organized as follows. Section 2 discusses the related work which mainly comprises the existing benchmark routing protocols. Section 3 explains the system model which includes network model and energy consumption model. Section 4 explains the proposed algorithm, which includes clustering methodology and data flow model. Section 5 describes the nine performance parameters which are used in simulations to measure and compare the performance of the proposed protocol. Results and discussion are presented in Section 6, with simulation environment and network configuration parameters. Finally, the conclusions and future work of this paper are given in Section 7.

## 2. Related Work

This section briefly describes the clustering hierarchical benchmark routing protocols proposed in the literature for homogeneous, two-tier, and three-tier heterogeneous WSNs. These benchmark protocols typically involve random deployment of SNs within the FoI to form a WSN. These protocols are based on the concept of rounds. Each round has two phases: (i) setup phase and (ii) steady-state phase. In the setup phase, the network is divided into clusters, CH are selected for each cluster from the SNs using a stochastic algorithm, and TDMA slots are assigned by the CH to its CMs. In the steady-state phase, CHs gather data packets from their CMs, aggregate the data packets, and send the aggregated data to the BS. The main difference in these benchmark routing protocols is the selection of CH in setup phase. These are discussed one by one, as follows.

1. LEACH [19] is proposed for homogeneous WSNs, where all the SNs have equal initial energy $E_{0}$. The CH is selected randomly from all the deployed *N* number of SNs who belong to a set *G* (i.e., set of those SNs which were not selected as CH in the previous $\frac{1}{p}$ rounds). All those SNs who belong to set *G* generate a random number $n_{i}$ between 0 and 1, and this number is compared with a threshold function T(N), i.e.,
  (1) $$T\left(N\right)=\left\{\begin{array}{cc} \frac{p}{1-p\left(r\;\mathrm{mod}\;\frac{1}{p}\right)}, & \;\forall \;N\;\epsilon \;G\\ 0, & \;\mathrm{otherwise}, \end{array}\right.$$
  where *N* is the number of deployed SNs, *p* is the predefined probability of CHs in SNs which is p=NumberofCHdevices/Numberoftotaldevices, and *r* is the current round number. An SN from set G becomes a CH if $\;n_{i}\leq T\left(N\right)$; otherwise, CM.
2. In LEACH-C [20], the CH is selected by the BS by considering the remaining energy and location of the SNs. At the end of each round, each SN sends information about its remaining energy and location to the BS. The BS calculates average energy based on the remaining energies of all the SNs. On the basis of the calculated average energy and locations of the SNs, CH is selected for each cluster by the BS.
3. TEEN [22] and LEACH use the same procedure for CH election, but in the case of TEEN, the CH sends the collected data from its CMs to the upper level of cluster (i.e., a cluster of CHs) which has its own CH that collects data and sends them to the next level of cluster. This process is repeated. At the end, only one CH is reporting to the BS. Furthermore, following the TEEN protocol, SNs sense their environment continuously but do not report until the sensed entity or value is greater than the hard threshold ($H_{t}$), which is a predefined value. Whenever the sensed value exceeds $H_{t}$, the SN triggers the transmitter to report the value. This is why TEEN sometimes misses some important information from the FoI.
4. IEE-LEACH [23] considers new threshold function with different probability function of CH selection. The new probability function depends on *p*, remaining and initial energy of SN which is candidate for CH, average and total energy of WSN, and total number of SNs in the FoI.
5. SEP [21] is the first protocol which considers the two-tier heterogeneity in WSN in terms of different initial energy for normal and advance SNs. Normal SNs have normal initial energy $E_{0}$, while advance SNs are equipped with relatively higher initial energy than $E_{0}$. SEP considers the optimal number of CHs, which is derived analytically. Furthermore, SEP defines two different threshold functions for two different types of SNs, i.e., normal and advance, with different probabilities of CH selection.
6. EEECA-THWSN [24] considers three-tier heterogeneity in WSNs which consists of standard, intermediate, and advanced SNs. Standard SNs have lower initial energy than intermediate SNs, and intermediate SNs have lower initial energy than advanced SNs. Furthermore, EEECA-THWSN proposes three different threshold function with there respective probabilities, for standard, intermediate, and advanced SNs.

Table 1 summarizes the major differences between proposed EEUCH and benchmark routing protocols. None of the benchmark protocols consider WuR-enabled SNs and they assume that as the SNs are deployed at FoI, the SNs start working (i.e., sense the surroundings, process the sensed data, and transmit them to CH). Moreover, in benchmark protocols, the BS is located within the FoI, and CHs are responsible for data reporting to BS. However, in the proposed protocol, we equipped SNs with WuR, enabling them to work in an on-demand manner. Furthermore, EEUCH is independent of the BS location and UAVs are responsible for data reporting to BS. The rounds of UAVs are application-dependent and there is no dependency among the rounds.

The authors in [30,34] proposed a hashing and partitioning approach for collecting data in WuR-enabled IoT applications that employs a scenario similar to ours, where UAVs are utilized for data collection. However, their work has notable differences and limitations. They divided SNs within the same cluster region of an UAV into different groups, and only one data packet is collected from each SN belonging to a specific group. Other SNs belonging to other groups in the same cluster region of the UAV coverage must wait for the next round of the UAV. The TDMA slot is shared with SNs through a hash function embedded in the broadcasted WuC, and the slot for data transmission assigned to an SN is based on the output of the hash function calculated by each SN. However, collisions can occur if two or more SNs produce the same output, resulting in multiple SNs using the same slot for data transmission. The likelihood of collisions increases with the number of SNs in a group. As only one packet per round of UAV per SN from a specific group is collected, this method increases latency and wastes UAV energy. SNs from the specific group with multiple packets must wait to transmit their packets for their next turn, and SNs belonging to other groups in the same cluster region must wait for subsequent rounds. Their system model in [30,34] is at layer 2 (MAC layer), whereas ours is at layer 3 (network layer). They eliminated overlapping of clusters using a complex partition algorithm, whereas the methodology proposed in this work differs significantly and is described in the following sections.

Based on the available literature work in this section, there is no hierarchical clustering routing protocol proposed for data collection from WuR-enabled ground SNs through UAVs that operates at the network layer. Therefore, we compare the proposed protocol with six benchmark routing protocols mentioned earlier, under the same scenario and network model, to evaluate its efficacy and assess its performance.

## 3. System Model

In this section, we will discuss the network model and energy consumption model to provide a detailed description of the system.

### 3.1. Network Model and Assumptions

**Figure 2:**
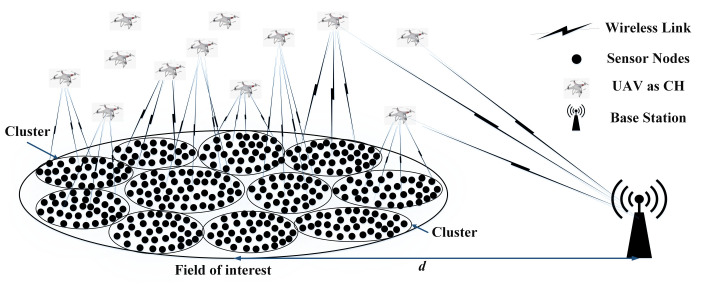
Proposed network model.

In our proposed model, *N* number of SNs (i.e., $S_{1},S_{2},S_{3},\ldots S_{N}$), each with the same $E_{0}$ (i.e., homogeneous WSN), are randomly deployed in the FoI which is located at a distance away from the BS, as shown in Figure 2.

Initially, all SNs in the FoI are in sleep mode (i.e., their MRs are turned off) to save energy by diminishing idle listening and overhearing. In sleep mode, the SNs sense their surroundings and wait for the WuC. When the UAVs arrive at a certain altitude from the FoI at their desired predefined hovering positions for data collection from SNs, they send a WuC to the ground SNs. The WuC is received by the WuRxs of the SNs. The SNs activate their MRs and send joining requests to the specific UAV whose WuC is detected by their WuRxs. It is critical for SNs to reliably transmit these joining requests in order for them to participate in cluster formation as CMs. When UAVs receive joining requests from SNs, they form clusters that include all of the SNs that sent joining requests. Hence, those SNs become CMs. In order to ensure reliable data communication, the UAV assigns TDMA slots to all of its CMs. These TDMA slots are then received by the MR of SNs. Within their assigned TDMA slots, data packets are transmitted from the CMs to the UAV, as each CM is assigned a unique TDMA slot to avoid any collisions or interference between them. After successful data communication, the UAV acknowledges the received data packets, CMs receive AcK from the UAV, and turn off their MR and enable sleep mode. It is noteworthy that the SNs deployed at the cluster boundaries may receive multiple WuCs from different UAVs. However, an SN can only respond to the first WuC received from a UAV; all subsequent WuCs received from other UAVs are ignored. Consequently, an SN can only be a CM of a single UAV in the particular round, which eliminates overlapping among the clusters. Once SNs receive their first WuC, all the clusters operate independently of one another. Furthermore, it is assumed that UAVs have enough energy to complete a single round (i.e., move to the FoI, make clusters there, collect data packets, and turn back) and the energy deficiency of the UAVs does not affect the continuity in data packet collection from ground SNs during a single round.

For the sake of clarity, the following assumptions are made:

1. All SNs have individually unique identification.
2. SNs and BSs are stationary.
3. SNs are not utilized again once dead, i.e., after full consumption of their battery.
4. All SNs must be equipped with WuR, i.e., they must have a WuRx with extremely low power consumption and MR for data packets transmission.

### 3.2. Energy Consumption Model

In this subsection, we discuss the radio energy consumption of an SN’s communication unit in the proposed system.

**Figure 3:**
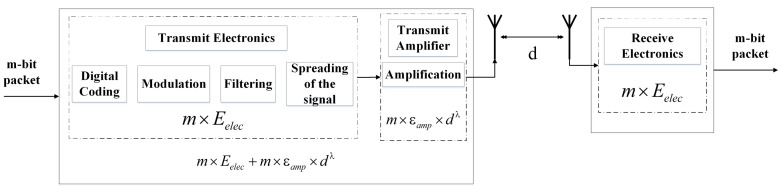
Radio energy consumption model.

In EEUCH, the communication link is between the SN and UAV, while in the existing benchmark protocols this link is between the SNs or between the SNs and BS. However, the proximity of the UAV to the SNs enables that the link between the UAV and the SNs can also use the IEEE 802.15.4 (ZigBee) and IEEE802.11ah (WiFi) [35] protocols. These protocols are medium range that could achieve transmissions several hundred meters long. These are the same technologies which are used in the existing benchmark routing protocols [29], so the radio model does not alter much on changing the channel from SN or BS to UAV. The hardware energy consumption model of an SN is shown in Figure 3.

The radio energy consumption of the SNs is due to their two major hardware components, i.e., (i) transmit electronics and (ii) transmit amplifier. The energy consumed by the transmit electronics is due to digital coding, modulation, filtering, and spreading of digital signal at transmitter, to transmit *m* number of bits, which is given as $m\times E_{elec}$. This energy consumption is the same for the receiver as well. Moreover, the energy consumed by transmit amplifier depends on the distance *d* between transmitter and receiver antennas, amplification factor $\varepsilon_{amp}$, and path loss exponent λ, to transmit *m* number of bits. It is given as $m\times \varepsilon_{fs}\times d^{\lambda }$. The minimum transmit energy consumed by the SN required to achieve acceptable received $E_{b}/N_{0}$ at the receiver (UAV) is given in (2) below, where $E_{b}$ is received energy per bit and $N_{0}$ is the noise power spectral density [13,19,20,21,22,23,24]. It is noteworthy that two separate channel models, free space ($d^{2}$) and multipath ($d^{4}$) as mentioned in Equation (2), are considered in this study. These simplified models are based on certain assumptions, as described in [36]. It is also important to mention that the benchmark and other routing protocols follow the same energy consumption model.

(2) $$E_{TX}=\left\{\begin{array}{c} mE_{elec}+m\varepsilon_{fs}d^{2},\;\mathrm{if}\;d\leq d_{0}\\ mE_{elec}+m\varepsilon_{mp}d^{4},\;\mathrm{if}\;d>d_{0} \end{array}\right..$$

In (2), $E_{TX}$ is the amount of energy consumed to transmit *m* number of bits, $E_{elec}$ is the amount of energy required for transmit electronics to process a single bit, parameter $\varepsilon_{fs}$ and $\varepsilon_{mp}$ are the amplification factors in case of free-space and multipath, respectively, and $d_{0}=\sqrt{\varepsilon_{fs}/\varepsilon_{mp}}$.

The energy consumed by the radio amplifier is significantly higher than the energy consumed by the transmit electronics. The difference in energy consumption between the two is directly related to the distance between the transmitter and the receiver antennas, given a specific channel model [18,19,23,24]. The efficient delivery of data is critical to prolonging the lifetime of a network. Therefore, this work proposes an energy-efficient routing protocol to achieve this goal.

## 4. Proposed Protocol

In this section, we present the methodology used for cluster formation and illustrate the data flow model of our proposed EEUCH protocol.

### 4.1. Cluster Formation

In this subsection, we describe a single round of the proposed EEUCH protocol, which involves dividing the deployed SNs into clusters, where each cluster has a UAV as a CH that collects data from its CMs and sends it to the BS. In each round, UAVs arrive at predefined hovering positions, at a certain altitude from the FoI, from which they can cover the maximum number of SNs. Since unlicensed spectrum is utilized, the UAVs perform CCA first to ensure that the unlicensed channel is free. Once CCA is successful, the UAVs broadcast WuCs to the ground SNs, initiating the cluster formation phase. After the clusters are formed, the SNs of each cluster become CMs of a particular UAV, and the steady-state phase starts, during which data are collected from the ground SNs. At the end of successful data collection, the CMs enable sleep mode. Hence, a single round is completed.

#### 4.1.1. Setup Phase

**Figure 4:**
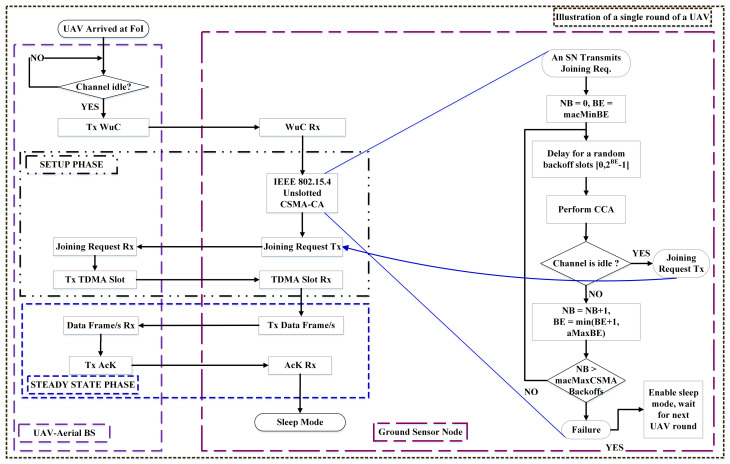
Block diagram of the proposed protocol.

We explain a single cluster formation here for a single UAV for a single round, as depicted in Figure 4. Since there is no overlapping among the clusters and they are independent, each cluster formation uses the same technique. At the start of each round, all ground SNs are in sleep mode so their MRs are in off state, but the WuRx, which consumes extremely low energy, is always on and waits for a WuC from a UAV. As the WuC is detected by the SN’s WuRx from an arrived UAV that has already performed successful CCA, it turns on its MR which takes a little time (called mode switching time (MST)) and performs CSMA-CA before sending a joining request in response to the particular UAV’s WuC. When the broadcasted WuC is received by multiple SNs, each SN wants to send a joining request individually in their active state. However, since the channel is shared among all the SNs, collisions in the joining requests from SNs in response to the WuC are inevitable. Thus, to avoid collisions we employ IEEE 802.15.4 nonbeacon mode unslotted CSMA-CA [37] to ensure the successful transmissions of joining request sent from SN to UAV, as illustrated in the figure. The successful transmission of this joining request is important, because on the basis of this request it is decided whether the SN takes part in cluster formation or not.

In unslotted nonbeacon mode CSMA-CA, each SN follows a back-off (BO) algorithm, which involves waiting for a random duration before performing CCA to check the channel’s status. If the channel is found to be idle, the SN proceeds to transmit its joining request. Conversely, if the channel is busy, the SN increments the back-off counter (NB) and adjusts the back-off exponent value accordingly. The SN then checks if the maximum back-off attempts (macMAXCSMABackoffs) have been reached. If the macMAXCSMABackoffs have been reached, the SN fails to transmit its joining request and is unable to participate in cluster formation. However, if the macMAXCSMABackoffs have not been reached, the SN initiates a new random back-off followed by CCA. This process continues until the SN successfully transmits its joining request or the maximum attempts are reached. This process is illustrated on the right side of Figure 4.

The probability of channel busyness after CCA (γ) is a key parameter in modeling the behavior of the CSMA-CA. In the existing literature, two mathematical frameworks have been used to analytically model γ. The first approach is based on the M/G/1 queue which considers an infinite number of packets with the SN, as discussed in [38]. The second approach is based on the M/G/1/2 queue which considers a maximum of two packets in the SN’s queue, as presented in [31]. In this study, we assume the M/G/1/2 analytical framework to calculate the probability that an SN is unable to join the cluster, indicating that it is dropped and unable to participate in cluster formation. This probability is mathematically expressed as

(3) $$P_{drop}=\gamma^{macMAXCSMABackoffs+1},$$

where $P_{drop}$ denotes the probability that an SN is dropped and unable to participate in the current UAV round’s cluster formation, and macMAXCSMABackoffs is the maximum number of CCA attempts, which is assumed as seven from the study [31]. When an SN fails to transmit its joining request, it enters sleep mode to conserve energy. It is important to note that this work does not encompass the modeling of CSMA-CA.

Parallel to this CSMA-CA process, the SN makes data packets ready to be transmitted. This process happens at each SN in the FoI, i.e., WuC detection by the SN’s WuRx, turning on the MR and making data packets, performing CSMA-CA, and sending a joining request. The UAV receives a joining request from each SN. After receiving a number of joining requests from the SNs, the UAV notes the IDentity (ID) of each particular individual SN, marks them as CMs, and assigns TDMA slots to these CMs. Thus, a cluster is formed with UAVs as CHs. This is called the setup phase, which is followed by the steady-state phase.

#### 4.1.2. Steady-State Phase

In the steady-state phase of EEUCH, each SN that has been selected as a CM has a TDMA slot assigned to it by its respective CH, which is the UAV. Each CM must transmit its data packets within its assigned slot. Once the data transmission is successful, the UAV sends an AcK to the ground SN. When the SN receives the AcK, the steady-state phase ends. When the CH acknowledges the data packets, the CM turns off its MR and enters sleep mode. This marks the completion of a single round. The duration of each round and the time between consecutive rounds depend on the application’s requirements.

Figure 4 illustrates the complete flow of a single round of a UAV in the proposed EEUCH routing protocol. The dotted lined box labeled “Illustration of a single round of a UAV” encloses the sequence of processes that occur during the round. The figure comprises two vertical boxes. The left vertical box, labeled “UAV-Aerial BS”, shows all the processes that occur at the UAV, including CCA and WuC transmission. The right vertical box, labeled “Ground Sensor Node”, demonstrates all the processes that occur at the SN, such as WuC reception, CSMA-CA, and joining request transmission. Additionally, the figure contains two horizontal boxes. The upper horizontal box, marked as “SETUP PHASE” in its top left corner, contains all the processes that occur during the setup phase, including cluster formation and TDMA slot allocation to the SNs. The lower horizontal box, marked as “STEADY STATE PHASE” in the bottom of its left corner, encloses all the processes that occur during the steady-state phase, such as data transmission to UAV and reception of AcK from the UAV. The figure demonstrates a single round of a UAV where all the required processes occur at the UAV and ground SNs are presented in distinct boxes. The cluster formation (setup phase) and steady-state phase are each enclosed in a distinct dotted box, representing the different stages of a single round of the proposed protocol.

### 4.2. Data Flow Model

The data are collected from the deployed SNs in their respective assigned TDMA slots. Each SN sends data packets to the specific CH, which is the UAV. We employ selective repeat automatic repeat request (ARQ) protocol for reliable data transmission [39]. In EEUCH, the SN will discard all data packets that have been sent to the UAV and are acknowledged by the UAV due to the SN’s memory constraints. For those data packets which are sent to the UAV but are not acknowledged yet, the SN must transmit these packets again in the subsequent round to UAV to ensure reliability.

The reliable transmission of data between SNs and a UAV is essential in WSNs. This reliable transmission is affected by various factors such as the duration of TDMA slot assigned by the UAV and the channel behavior. It is essential to ensure that the channel conditions remain stable for reliable data transmission. If the channel conditions change frequently, the probability of receiving AcK of data packets from the UAV is reduced, leading to longer waiting time for the SN and higher energy consumption. Additionally, in such scenarios, the SN retains the data packets for the next round of UAV, and no further sensed data can be stored because of the limited memory capacity of the SN.

The duration of the TDMA slot assigned by the UAV is another vital factor that must be considered to ensure reliable data transmission. If the TDMA slots have a short duration, not all data packets can be reliably communicated within that period. As a result, the remaining data packets will have to wait for the next round of UAV. Therefore, the TDMA slot duration should be set so that all data packets can be communicated reliably during the assigned slot. However, if the TDMA slots have a more extended duration, the UAV hover time increases.

**Figure 5:**
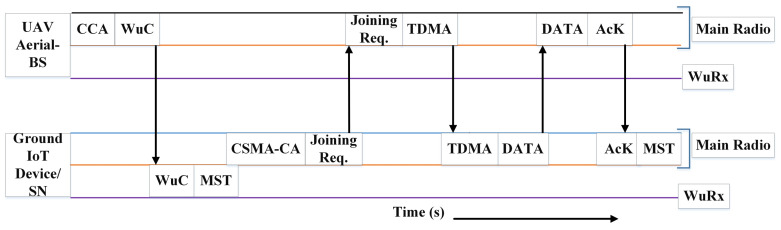
Illustration of the data flow for EEUCH.

In summary, for reliable data transmission, it is crucial to carefully tune the parameters such as the TDMA slot duration, channel condition, and UAV hover time. By optimizing these parameters, the reliability of data transmission can be improved, and the SN can efficiently transmit the sensed data to the UAV without experiencing significant delays or data losses. The whole operation procedure of the EEUCH protocol is depicted in Figure 5.

## 5. Network Performance Parameters

In this section, we present and define nine performance parameters that are used in this paper to comprehensively evaluate and compare the effectiveness of the proposed EEUCH protocol against existing benchmark routing protocols. These performance parameters are carefully selected to assess various aspects of the proposed protocol’s performance and facilitate informed comparisons with existing benchmark routing protocols.

1. Number of alive SNs: This parameter represents the number of SNs having residual energy ($E_{r}$) greater than zero, known as alive SNs, as the rounds pass. Every single data packet transmission consumes a unit energy, and as several data packets are transmitted in a single round from SN to UAV, the SN energy is reduced from initial energy to zero as the rounds pass. This parameter provides an important metric of how many SNs are still operational at a particular round of the protocol. The higher the number of alive SNs, the better the protocol’s efficiency in energy conservation, prolonging the network lifetime.
2. Number of dead SNs: This parameter indicates the number of SNs whose residual energy drops to zero during the rounds of the protocol. As each data packet transmission consumes a unit of energy, the SNs gradually consume their initial energy and eventually become unable to transmit data. This parameter indicates the number of SNs that have failed and cannot participate in subsequent rounds.
3. Total number of packets collected from FoI: This parameter determines the total number of data packets collected from the FoI over the course of the network’s lifetime. This metric demonstrates the overall effectiveness of the protocol in gathering data. A more efficient protocol should be able to collect a larger number of data packets while extending the network lifetime.
4. Remaining network energy over the rounds: This parameter quantifies the amount of network energy that remains as the rounds pass. It reflects the overall network sustainability, which is vital for the longevity of the network lifetime. It is calculated by subtracting the total energy consumed till the current round from the initial network energy. A more efficient protocol should achieve a longer network lifetime by minimizing the rate at which energy is consumed. The total network initial energy is different for homogeneous, two-tier, and three-tier heterogeneous WSNs. For a homogeneous network, initial energy can be calculated using (4) below for which LEACH, LEACH-C, TEEN, IEE-LEACH, and the proposed EEUCH routing protocols are employed, while for two-tier and three-tier heterogeneous networks, we used (5) and (6), in which SEP and EEECA-THWSN routing protocols are employed, respectively. Equations (4)–(6) are given, respectively, below.
  (4) $$E_{homo}^{N}=N\times E_{0},$$
  where $E_{homo}^{N}$ denotes the total homogeneous network initial energy, *N* is the total number of SNs, and $E_{0}$ is the standard initial energy for an SN.
  (5) $$E_{SEP}^{N}=E_{0}\times \left(N_{nrm}+\left(1+\alpha_{SEP}\right)\times N_{adv}\right),$$
  where $E_{SEP}^{N}$ is the total initial energy for a two-tier heterogeneous network, $N_{nrm}$ is the number of normal SNs which have initial energy as $E_{0}$ each, and $N_{adv}$ is the number of advance SNs which have initial energy as $\alpha_{SEP}$ times $E_{0}$ each. $N_{nrm}$ and $N_{adv}$ can be calculated using the following equations:
  $$N_{nrm}=N\times \left(1-m_{SEP}\right),$$
  and
  $$N_{adv}=N\times m_{SEP},$$
  where $m_{SEP}$ is the percentage of $N_{adv}$ in *N*. Furthermore,
  (6) $$E_{EECA}^{N}=E_{0}\times \left(N_{std}+\left(1+\alpha \right)\times N_{int}+\left(1+\beta \right)\times N_{advc}\right),$$
  where $E_{EECA}^{N}$ is the total initial energy for a three-tier heterogeneous network, $N_{std}$ is the number of standard SNs having initial energy $E_{0}$ each, $N_{int}$ is the number of intermediate SNs having initial energy α times $E_{0}$ each, and $N_{advc}$ is the number of advance SNs having initial energy β times $E_{0}$ each. $N_{std}$, $N_{int}$, and $N_{advc}$ can be calculated as
  $$N_{std}=N\times \left(1-m_{i}-m_{a}\right),$$
  $$N_{int}=N\times m_{i},$$
  and
  $$N_{advc}=N\times m_{a},$$
  where $m_{i}$ and $m_{a}$ are the percentages of $N_{int}$ and $N_{advc}$ in *N*.
5. Average network energy reduction: This shows the decrement in the average energy of the network. A better routing protocol should have slow and linear decrement of average network energy over the rounds. Average energy of the network can be calculated as
  (7) $$E_{avg}^{N}=\frac{E_{r}^{N}}{N},$$
  where $E_{r}^{N}=E_{init}^{N}-E_{c}^{N}.$ Here, $E_{r}^{N}$ shows the remaining energy of the total network in a particular round, $E_{c}^{N}$ is the energy consumed till the previous round is completed, and $E_{init}^{N}$ is the initial energy of the network which is different in each case of homogeneous, two-tier, and three-tier heterogeneous networks.
6. Network energy consumed: This parameter shows the cumulative energy consumption of the network over the rounds. As SNs die and their number decreases with the rounds, the energy consumption decreases. This parameter indicates the accumulated energy consumed by each alive SN and CH node till the previous round was completed. Since the number of alive SNs and CHs in each round varies, there is no fixed formula to calculate the value of $E_{c}^{N}$. However, network energy consumed in a single round can be calculated as
  (8) $$E_{c}^{R}=E_{TX}\times \left(N_{alive}-CH_{nodes}\right)+\left(E_{TX}+E_{DA}\right)\times CH_{nodes},$$
  where $E_{c}^{R}$ is the energy consumed in a single round, $E_{TX}$ is given in Equation (2), $N_{alive}$ is the total number of alive SNs in the particular round, $CH_{nodes}$ CH nodes selected from $N_{alive}$, and $E_{DA}$ is data aggregation energy consumed by CH node.
7. Network lifetime: This is the time from the start till all the SNs die. More precisely, it is the time taken from the start (i.e., WSN or SNs deployment) till the last SN’s death. Thus,
  (9) $$T_{N}=\left(t_{r}\right)\times \left(ASD\right)-\left(T_{START}\right),$$
  where $T_{N}$ is network lifetime, $t_{r}$ is the time in which the data transmission occurs in each round, “ASD” is the number of rounds when all SNs die, and $T_{START}$ is the time when the WSN starts operating.
8. Stability time: It refers to the duration of time from the start of the network operation until the first SN in the network dies due to energy depletion. Stability time is an important metric to consider because it gives an indication of the overall network stability and robustness. A longer stability time means that the network is able to sustain its operation for a longer period without any significant disruptions or failures. This is particularly important for applications where the WSN is deployed in remote or inaccessible locations, and maintenance or repair may not be feasible or cost-effective. Stability time can be written as
  (10) $$T_{S}=\left(t_{r}\right)\times \left(FSD\right)-\left(T_{START}\right),$$
  where $T_{S}$ is the stability time and “FSD” is the number of rounds when first SN dies.
9. Average throughput: It is an essential performance parameter that provides a measure of the amount of data successfully collected from the FoI per unit time in WSNs. A higher average throughput indicates that the protocol is more efficient and effective in delivering data from the FoI. This parameter is crucial for various applications, especially in real-time monitoring and control systems, where timely delivery of data is critical. Therefore, the evaluation and comparison of average throughput among different WSN routing protocols can provide valuable insights into the network’s performance. Average throughput can be calculated as
  (11) $$T_{avg}=\frac{Number\;of\;packets\;received\times Packet\;size}{Number\;of\;rounds}.$$
  Here, $T_{avg}$ represents the average throughput of the network.

## 6. Simulations Results and Discussions

**Figure 6:**
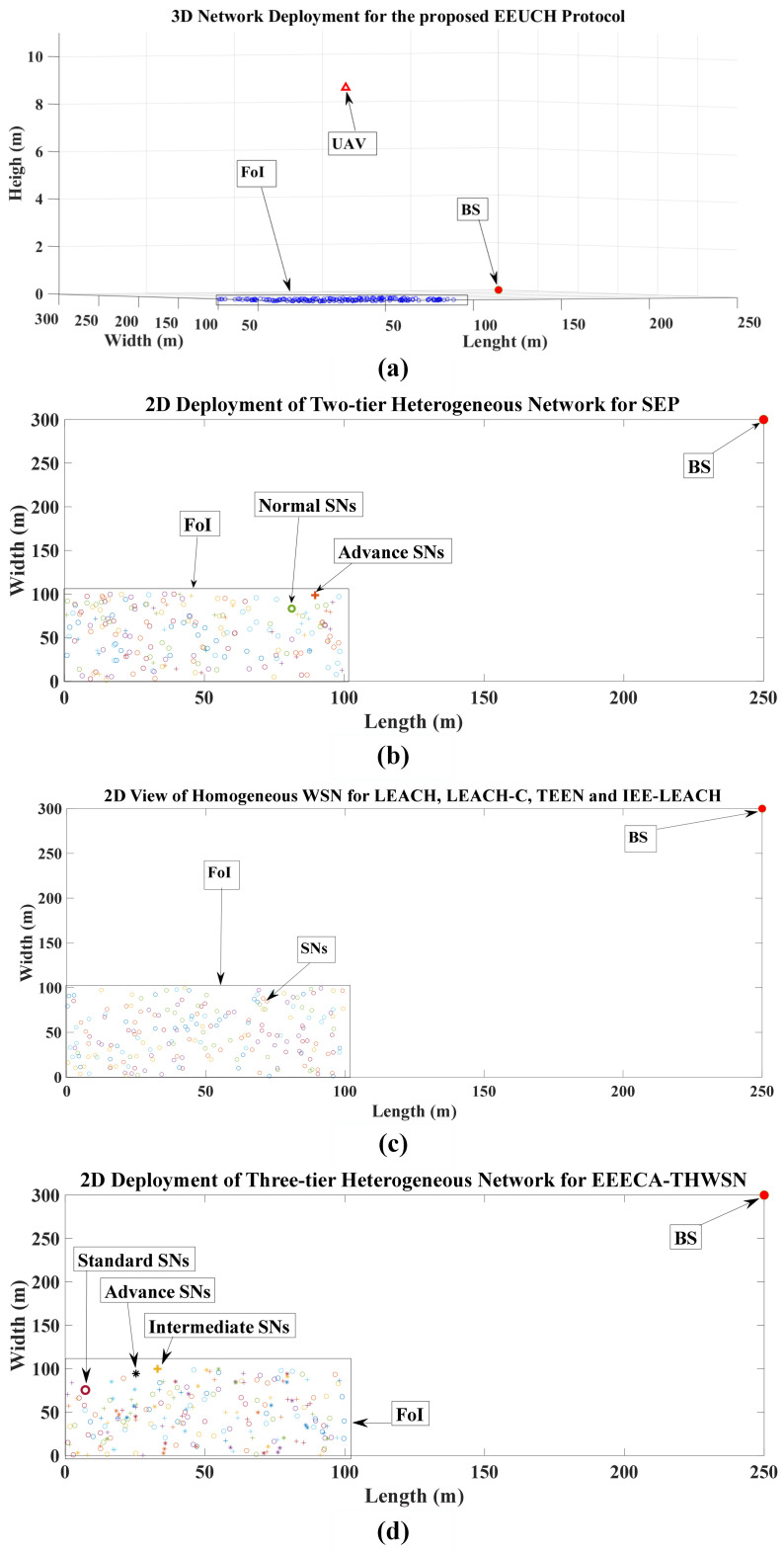
Geographical locations of FoI, UAV-ABS, SNs, and BS are shown for our proposed EEUCH and other benchmark protocols simulated in MATLAB 2021b: (**a**) 3D view of EEUCH, (**b**) 2D view of SEP: the two-tier heterogeneity concept is shown, (**c**) 2D deployment of homogeneous WSNs i.e., LEACH, LEACH-C, TEEN, and IEE-LEACH, and (**d**) 2D deployment of three-tier heterogeneous WSNs, i.e., EEECA-THWSN.

In this section, we present the performance of the proposed EEUCH protocol compared with the existing benchmark routing protocols, i.e., LEACH [19], LEACH-C [20], TEEN [22], SEP [21], IEE-LEACH [23], and EEECA-THWSN [24]. In simulation experiments, we consider that a WSN of N=200 SNs is deployed in the FoI which is 250 m away from the BS in a 100 m × 100 m area. For the simplicity and better comparison of EEUCH with the benchmark routing protocols, we simulate EEUCH for a single cluster. The UAV does not change its position much during the steady-state phase of a round to affect the channel conditions. Considering the mobility nature and error in the exact deployment of UAV, we assume flexibility up to 2 m on each axis of UAV deployment [29]. It is important to note that the same results are obtained for larger areas (FoIs) in proportion to the number of SNs and UAVs, i.e., two UAVs will cover twice the area and twice the number of SNs, due to the cluster independence and no overlapping. MATLAB 2021b was chosen as the simulation tool for our system primarily due to the availability of toolboxes that support wireless communication and network protocols. Its user-friendly environment facilitates smooth algorithm implementation, simulation, and result analysis. MATLAB’s ability to generate high-quality figures significantly enhances the visualization of simulation results. Furthermore, its computational efficiency makes it a highly favorable choice when compared to other simulation environments. We run the simulation for 3000 rounds. The geographical three-dimensional locations of the WSN, SNs, BS, and UAV are shown in Figure 6. The predefined network configuration parameter values are given in Table 2.

### 6.1. Number of Alive Sensor Nodes over the Rounds

**Figure 7:**
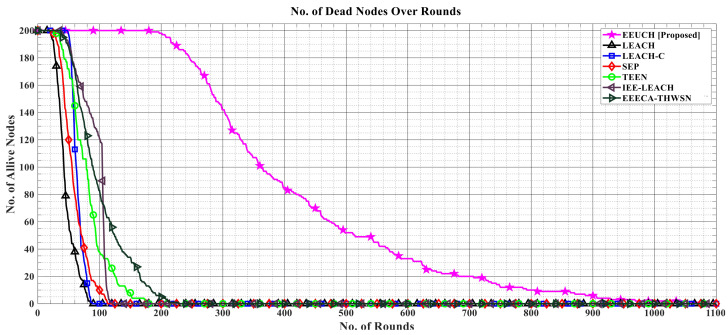
A comparative analysis of the number of alive SNs over the number of rounds.

In Figure 7, the number of alive SNs over the number of rounds is shown, with all two hundred SNs initially in an active state, meaning that they have energy. The graphs display a decreasing trend as the number of rounds increases, indicating the gradual decrease in the number of SNs due to energy depletion. The proposed protocol, EEUCH, outperforms the benchmark routing protocols, as it results in a larger number of alive SNs for a longer time (i.e., number of rounds). This improvement is attributed to the shorter transmission distance between the ground SNs and UAV, as well as the elimination of additional energy consumption by CH SN. This leads to a decrease in the overall energy consumption, which in turn, and consequently, leads to longer lifetimes of the SNs.

### 6.2. Number of Dead Sensor Nodes over the Rounds

**Figure 8:**
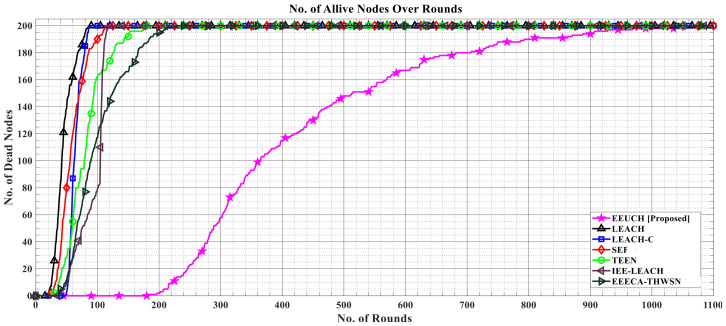
A comparative analysis of dead SNs over the number of rounds.

Figure 8 illustrates how many of the SNs die over the rounds. The y-axis represents number of dead SNs while the number of rounds are taken along the x-axis. As we move along the x-axis, the number of deaths increases. In EEUCH, the last SN lives till 1080 rounds, while in LEACH, LEACH-C, SEP, TEEN, IEE-LEACH, and EEECA-THWSN, the last SN dies at round number 86, 89, 113, 180, 121, and 229, respectively. This clearly indicates the proposed protocol’s effectiveness in enhancing network life, due to the elimination of CH from SNs, as CH consumes more energy than CMs. In the proposed EEUCH protocol, UAV acts as CH and all SNs are CMs.

### 6.3. Remaining Network Energy over the Rounds

**Figure 9:**
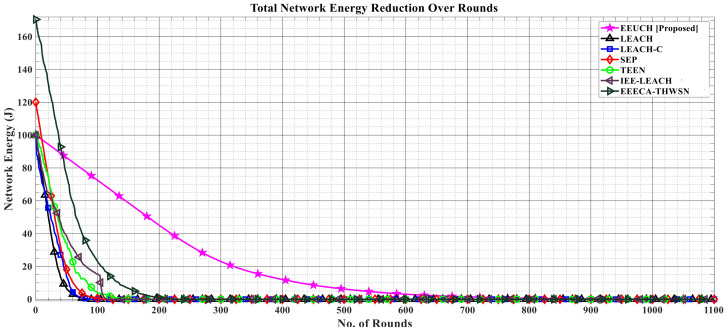
Reduction in the total energy of the network over the number of rounds.

Figure 9 presents the remaining network energy over the rounds. Along the y-axis we have the total initial energy of the WSNs (which is different for homogeneous, two-tier, and three-tier heterogeneous WSNs). As we move along the x-axis (rounds spent), the network energy is reduced. Due to the heterogeneous nature of WSNs in the case of EEECA-THWSN and SEP protocols, they have more initial network energy than the others, but their performance is still significantly lower than our proposed EEUCH protocol. This is because in EEUCH the UAV can move dynamically towards the ground SNs for data packets’ collection, while in the case of the benchmark protocols the data packets are transmitted by the CH to the BS (located at longer distance from the CH).

### 6.4. Total Network Energy Consumption over the Rounds

**Figure 10:**
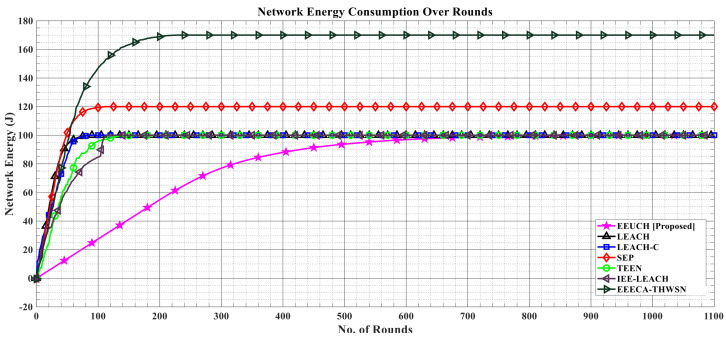
Total energy consumption over the number of rounds.

Figure 10 depicts the network energy consumption over the number of rounds. In the benchmark protocols, data packets are transmitted from SNs to the CH in each cluster and then from each CH to the BS, which means that more transmissions occur for a single data packet in the FoI between the SNs, resulting in more network energy consumption, whereas in EEUCH, once the data packet is transmitted from SNs to UAV, no other transmission occurs between the SNs or in WSN. As a result, our proposed EEUCH uses the lowest amount of energy in each round.

**Figure 11:**
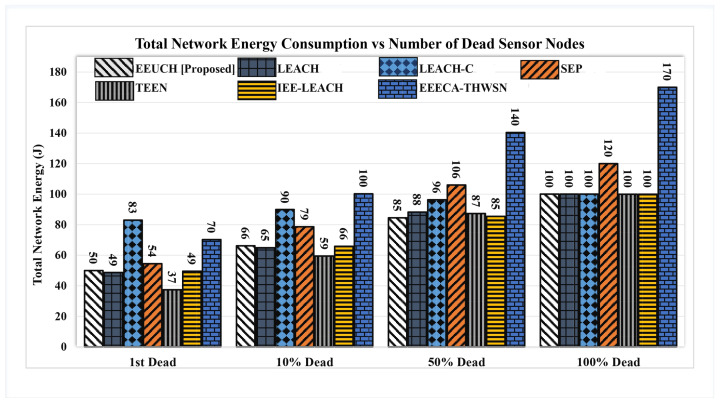
Network energy consumption versus percentage of node deaths.

Figure 11 shows the amount of total network energy consumption when the first SN dies, and ten, fifty, and one hundred percent of SNs die. The number mentioned above a bar represents the amount of energy consumed in joules with the respective network condition along the x-axis. When the first SN dies, 50 joules out of 100 joules energy is consumed in EEUCH, which means almost half of the network energy is consumed. TEEN performs better, for which the 37 joules energy is consumed out of 100 joules. However, when fifty percent of SNs die, the consumed energy of EEUCH and IEE-LEACH is equal while the remaining protocols have consumed much more energy, which means that the SNs die approximately linearly as the number of rounds increases.

### 6.5. Network Average Energy Reduction over the Rounds

**Figure 12:**
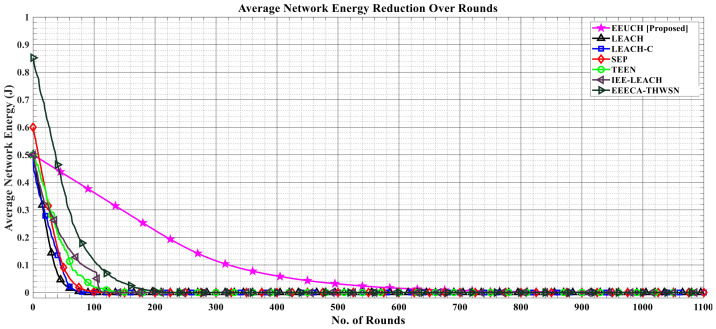
Reduction in the average energy of the network over the number of rounds.

Figure 12 depicts how the network average energy decreases over the rounds. The figure clearly illustrates that for the proposed EEUCH, the average network energy decreases almost linearly with the rounds spent. This is because of the fact that each SN sends data packets to the UAV, which is relatively closer than the BS, and hence the transmission energy is reduced. In the case of benchmark protocols, the CH is responsible for sending data packets collected from SNs to the BS. Initially, all SNs have enough energy, but as rounds are spent, the energy of the SNs decreases, and a stage is reached when the elected CH from SNs has very little energy, preventing it from transmitting data packets to the BS; thus, the CH dies during the round, resulting in nonlinearity in the graphs.

**Figure 13:**
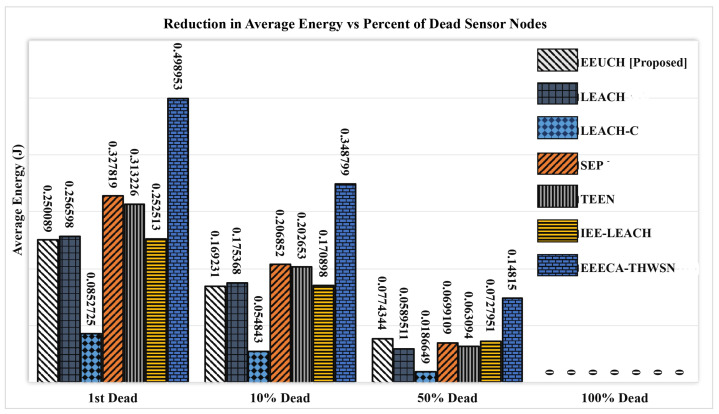
Reduction in the network average energy versus percentage of node deaths.

The amount of total network average energy reduction when first SN dies, ten, fifty, and one hundred percent of SNs die is shown in Figure 13. The amount of average network energy reduced from the total initial network energy is represented by the number mentioned above a bar. The linear consumption of network energy for the EEUCH can also be observed in this figure. Furthermore, when the first SN dies, the energy of the EEUCH is much lower, indicating lower performance of the proposed EEUCH, but when half of the SNs die, the EEUCH shows the best performance among the benchmark protocols. This is because CHs died prematurely in benchmark protocols.

### 6.6. Total Number of Packets Collected over the Rounds

**Figure 14:**
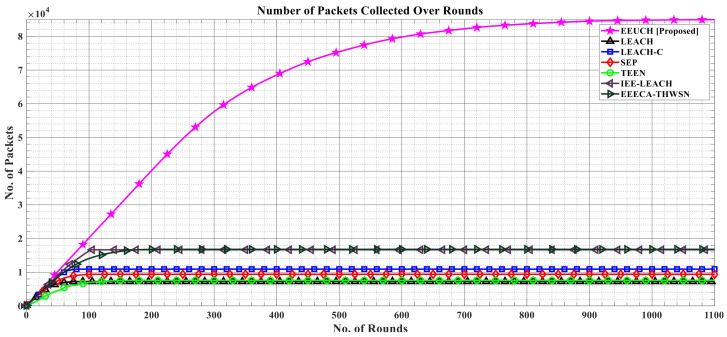
Total number of packets collected from the FoI over the number of rounds.

**Figure 15:**
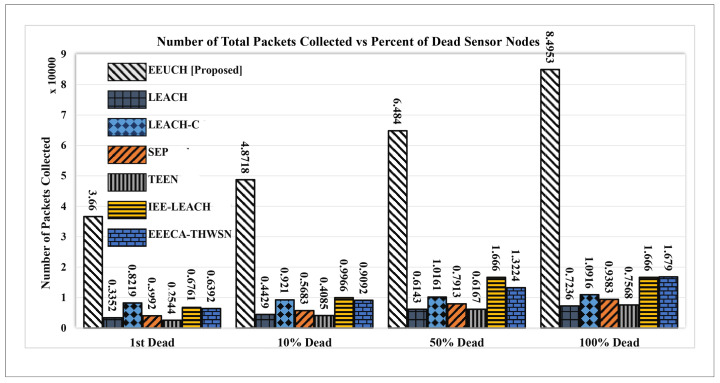
Number of packets collected versus percentage of node deaths.

Figure 14 illustrates the cumulative number of packets collected from the FoI by the EEUCH protocol as the rounds progress and the SNs consume energy. An SN can sense its surroundings and share the sensed information if it has sufficient energy. As discussed earlier, EEUCH protocol consumes energy linearly, resulting in a longer network lifetime, which allows the SNs to remain active for a longer period of time. This prolonged lifetime results in a greater amount of data being collected from the FoI as the SNs have energy to sense and share information. In contrast, other benchmark routing protocols consume energy nonlinearly due to the early death of CH, leading to a shorter network lifetime and less data collection from the FoI. Figure 15 provides further evidence of the EEUCH protocol’s ability to collect more data by showing the total number of packets gathered from the FoI as the number of dead SNs increases, i.e., at the first SN death, ten, fifty, and one hundred percent of SNs’ death. The total number of packets collected is indicated above each bar.

### 6.7. Network Stability Time

**Figure 16:**
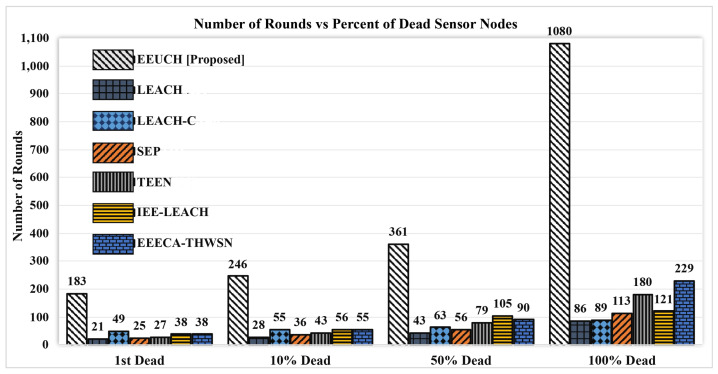
Number of rounds versus percentage of node deaths.

Stability time is the time taken by the network from the start till the first SN dies. Stability time indicates the time for which all the SNs are responding, i.e., all the SNs are alive. It depends upon the time period of each round and the number of rounds till the death of first SN. Stability time for all the benchmark and proposed protocol is given in Table 3. It can be seen from the table that EEUCH outperforms EEECA-THWSN, IEE-LEACH, TEEN, SEP, LEACH-C, and LEACH in terms of stability, with factors of 4.82, 4.82, 6.78, 7.32, 3.73, and 8.71, respectively. After round number 183, 21, 49, 25, 27, 38, and 38, the first SN dies in the case of using EEUCH, LEACH, LEACH-C, SEP, TEEN, IEE-LEACH, and EEECA-THWSN routing protocols, respectively, as shown in Figure 16. Furthermore, the time in seconds given in the table represents the time for which the WSN is sensing as well as transmitting the sensed data packets.

### 6.8. Network Lifetime

The network lifetime is a crucial performance metric for evaluating the effectiveness of any WSN routing protocol. Network lifetime refers to the duration between the deployment of the WSN and the time when all the SNs die due to energy depletion. Figure 16 depicts the number of rounds versus the percentage of SNs dying for both the proposed EEUCH protocol and the benchmark protocols. WSNs utilizing the EEUCH protocol exhibit a longer lifetime compared to the benchmark protocols. This is due to the fact that the proposed protocol consumes less energy, resulting in the efficient use of the available energy source (i.e., battery) of the SNs. Furthermore, Table 3 presents the lifetime performance of the benchmark protocols and the proposed protocol.

### 6.9. Average Network Throughput

**Figure 17:**
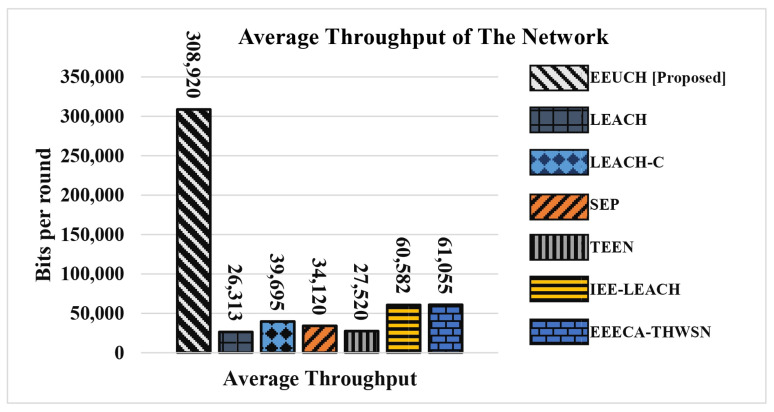
Average throughput.

The performance of different routing protocols in terms of average throughput is demonstrated in Figure 17, where the average throughput is defined as the average number of bits collected from the FoI in each round. As depicted in the figure, the proposed EEUCH protocol outperforms the other benchmark protocols significantly due to the prolonged life of SNs. In contrast, the benchmark protocols exhibit early SN death, resulting in lower throughput compared to the EEUCH protocol. It is worth mentioning that the EEUCH protocol efficiently utilizes the energy of each SN, which leads to a longer network lifetime. The longer SN lifespan ultimately leads to a more reliable and efficient data collection process, thus improving the overall performance of the proposed protocol.

### 6.10. Critical Discussion

In this manuscript, we proposed a novel clustering and routing protocol for UAV-based data collection from SNs. Our approach addresses cluster overlapping and utilizes UAVs as CHs for efficient data packet collection. We introduced a unique mobile CH technique for energy-efficient cluster formation without a setup phase. Our routing protocol extends to the MAC layer to enhance network reliability. We developed an innovative data flow model that prioritizes reliability and reduces the SN’s energy consumption. Additionally, our protocol incorporates WuRs to reduce SN energy consumption during communication.

The proposed protocol exhibits outstanding performance in several key aspects. Results from Section 6.1 and Section 6.2 reveal a substantial increase in SNs’ life span, with the proposed protocol extending their survival by 12.55, 12.13, 9.55, 6, 8.92, and 4.72 times compared to LEACH, LEACH-C, SEP, TEEN, IEE-LEACH, and EEECA-THWSN, respectively. Additionally, employing the EEUCH protocol allows SNs to maintain their energy up to 1080 rounds, significantly outperforming other protocols where SNs perish as early as round 86, 89, 113, 180, 121, and 229. Moreover, the EEUCH protocol excels in packet collection, gathering 11.74, 7.78, 9.05, 11.22, 5.10, and 5.06 times more packets compared to LEACH, LEACH-C, SEP, TEEN, IEE-LEACH, and EEECA-THWSN, respectively. The protocol also demonstrates improved network stability and extended lifetime, as evidenced by the evaluation in Table 3. Furthermore, the EEUCH protocol achieves better average throughput, transmitting 11.74, 7.78, 9.05, 11.22, 5.11, and 5.06 times more bits per round compared to LEACH, LEACH-C, SEP, TEEN, IEE-LEACH, and EEECA-THWSN, respectively.

Control overhead is a significant performance metric that measures the additional signaling and communication overhead associated with various operations in a protocol. This includes tasks such as cluster formation, CH selection, and coordination between CHs and member SNs. Control overhead encompasses the exchange of control messages such as cluster setup messages, join requests, CH advertisements, and cluster synchronization messages. Our proposed EEUCH protocol introduces certain elements that contribute to this metric. These include WuC reception, joining request transmission, TDMA frame reception and recognition of dedicated slots, and AcK reception for transmitted data. To quantify the control overhead generated by EEUCH, we consider sizes of 4000, 88, 32, 160, and 160 bits for data, AcK, WuC, join request, and TDMA slots allocation frame, respectively. The data size is taken from our benchmark routing protocols, as indicated in Table 2. The sizes of WuC and ACK are obtained from [31]. The sizes of joining request and TDMA slots allocation frames are assumed to be the same as those of request to send (RTS) and clear to send (CTS) frames in the IEEE 802.11 standard [40]. The assumption is made on the basis that the information carried by these frames are identical. This includes details such as the source and destination addresses, duration of the intended transmission, and a sequence number. The control overhead can be expressed as follows:

(12) $$Control\;Overhead=\frac{Total\;size\;of\;the\;control\;packets}{Total\;size\;of\;the\;data\;packets}\times 100,$$

where totalsizeofthecontrolpackets refers to the cumulative size of all control packets transmitted in the network per round, and the totalsizeofthedatapackets refers to the size of all data packets transmitted, per round. The total control overhead generated by the proposed EEUCH protocol is 11% in a single UAV round. This calculation takes into account that each SN transmits only one data packet in a single round.

The information coverage ratio is another significant metric that evaluates the effectiveness of SNs deployment in covering the monitored area within a WSN. It assesses the extent to which the deployed SNs capture and monitor the target FoI. In our proposed EEUCH protocol, the SNs are randomly deployed, and thus our focus shifts to the information coverage of the UAV, which represents the number of SNs covered by the UAV. Our goal is to maximize the information coverage by strategically deploying 200 SNs within the FoI. As mentioned in Table 2, the protocol ensures that the UAV covers a 100 × 100 m${}^{2}$ area by utilizing these 200 deployed SNs in a single sweep.

## 7. Conclusions and Future Work

In this article, we proposed an energy-efficient and reliable solution for data collection from WuR-enabled SNs deployed in remote areas using UAVs. Our proposed protocol, EEUCH, incorporates essential features such as WuR-enabled SNs, clear channel assignment, CSMA-CA, and TDMA slots, to ensure energy-efficient and reliable data collection. Additionally, EEUCH mitigates the issue of cluster overlapping. The use of WuR-enabled SNs facilitates energy-efficiency, while the incorporation of CSMA-CA and TDMA improves the reliability and efficiency of data transmission between SNs and UAVs. Overall, EEUCH is a promising solution for energy-efficient and reliable data collection in WSN-based IoT applications.

In our proposed model, randomly deployed SNs are equipped with MR and WuRx. The EEUCH routing protocol operates in rounds, where each round commences upon the arrival of UAVs at the FoI. The arrived UAVs trigger the MRs of SNs by broadcasting WuCs, make clusters of SNs, and reliably collect data from the cluster-member SNs using a novel data collection model, and the collected data are then forwarded to the BS. Thus, a single round of EEUCH is completed. Each cluster operates independently in every round of EEUCH once the SNs have received the WuCs.

In this study, we conducted a performance evaluation of our proposed EEUCH routing protocol and compared it with well-known benchmark routing protocols, such as LEACH, LEACH-C, SEP, TEEN, IEE-LEACH, and EEECA-THWSN, which are designed for homogeneous, two-tier, and three-tier heterogeneous WSNs. Our comparison results demonstrate that the EEECA-THWSN protocol performs better among the benchmark protocols. Therefore, we focus our comparison on EEUCH and EEECA-THWSN in this conclusion. The results show that the initial network energy consumption of EEECA-THWSN is 1.7 times higher than that of the proposed EEUCH protocol. By implementing EEUCH, SNs in the FoI can remain active for 4.72 times more rounds, collect 5.06 times more data packets, each containing 4000 bits, and achieve 7.956 times greater stability compared to the EEECA-THWSN routing protocol.

Considering the noteworthy enhancements in all the considered nine network parameters that were evaluated, the proposed model still faces certain challenges such as the efficient deployment of UAVs, determining the optimal number of UAVs to be used, determining the maximum number of SNs that can be covered by a single UAV, and addressing the energy constraints on UAVs during a single round. Moving forward, our future research will encompass a comprehensive analysis of the EEUCH protocol and six benchmark protocols, with a specific emphasis on evaluating control overhead and information coverage ratio. These areas will be the primary focus of our forthcoming research endeavors.

## Figures and Tables

**Table 1:** Comparative analysis of the proposed EEUCH and benchmark routing protocols.

Metric/Features	EEUCH [Ours]	LEACH [19]	LEACH-C [20]	TEEN [22]	IEE-LEACH [23]	SEP [21]	EEECA-THWSN [24]
Network scenario	Homogeneous	Homogeneous	Homogeneous	Homogeneous	Homogeneous	Two-tier heterogeneous	Three-tier heterogeneous
Cluster formation and CH election	Each UAV announces itself as a CH for its respective cluster	SNs decide CHs for each cluster randomly	BS decides CHs for each cluster by considering SN’s energy and location	SNs decide CHs for each cluster randomly, also clusters of CHs are formed	SNs decide CHs for each cluster by considering average and total energy of the WSN, residual energy of SN, and round number	SNs decide CHs for each cluster by considering the heterogeneity in SN’s energy	SNs decide CHs by considering the heterogeneity in SN’s energy, current and initial energy of WSN, average distance from BS and SN’s location
Location of BS	*d* distance away from the FoI	In the middle of FoI	In the middle of FoI	In the middle of FoI	In the middle of FoI	In the middle of FoI	In the middle of FoI
Simulation software	MATLAB	MATLAB	NS-2	MATLAB	MATLAB	MATLAB	MATLAB
WuR for SNs	Yes	No	No	No	No	No	No
Data collection from FoI	Whenever required (Application dependent)	Continuous	Continuous	Continuous	Continuous	Continuous	Continuous

**Table 2:** Initial network parameters [19,20,21,22,23,24,26,27,29].

Parameter Symbol	Description	Value	Unit
$E_{0}$	Initial energy of an SN	0.5	J
M×M	Network size	100×100	m${}^{2}$
*N*	Number of SNs	200	NA
*p*	CH percentage in N	0.2	NA
$r_{max}$	Max. number of rounds	3000	NA
*m*	Packet size	4000	bits
$E_{TX}$	Transmit electronics energy	$50\times 10^{-9}$	J/bit
$E_{RX}$	Receive electronics energy	$50\times 10^{-9}$	J/bit
$\varepsilon_{fs}$	Amplification factor of transmitting circuit for free space channel model	$10\times 10^{-12}$	J/bit/m${}^{2}$
$\varepsilon_{mp}$	Amplification factor of transmitting circuit for multipath channel model	$0.0013\times 10^{-12}$	J/bits/m${}^{4}$
$d_{0}$	Threshold distance	87.07058	m
$E_{DA}$	Data aggregation energy	$5\times 10^{-9}$	J/bit/signal
(x,y)	BS location	[250,300]	[m, m]
$\alpha_{SEP}$	Coefficient of ($E_{0}$) for $N_{adv}$	2	NA
α	Coefficient of ($E_{0}$) for $N_{int}$	2	NA
β	Coefficient of ($E_{0}$) for $N_{advc}$	3	NA
$m_{SEP}$	Probability of $N_{adv}$	0.2	NA
$m_{i}$	Probability of $N_{int}$	0.3	NA
$m_{a}$	Probability of $N_{advc}$	0.2	NA
*n*	Number of UAVs	1	NA
*h*	Height of UAV	8–10	m

**Table 3:** Stability and lifetime of the network based on different routing protocols.

Protocol	Network Stability Time	Network Lifetime
LEACH [19]	420 s	$1.72\times 10^{3}$ s
LEACH-C [20]	980 s	$17.8\times 10^{3}$ s
SEP [21]	500 s	$2.26\times 10^{3}$ s
TEEN [22]	540 s	$3.6\times 10^{3}$ s
IEE-LEACH [23]	760 s	$2.42\times 10^{3}$ s
EEECA-THWSN [24]	760 s	$4.58\times 10^{3}$ s
EEUCH [Proposed]	$3.66\times 10^{3}$ s	$21.6\times 10^{3}$ s

## Data Availability

Not applicable, as all the results are based on simulation data.

## Abbreviations

A list of the abbreviations used in this paper is given as follows:

**AcK**	Acknowledgment
**ARQ**	Automatic repeat request
**ASD**	Number of rounds spent when all SNs die
**BE**	Backoff exponent
**BS**	Base station
**CH**	Cluster-head
**CCA**	Clear channel assignment
**CMs**	Cluster members
**CSMA-CA**	Carrier sense multiple access/collision avoidance
**CTS**	Clear to send
**EEECA-THWSN**	Enhanced energy-efficient clustering approach for three-tier heterogeneous
	wireless sensor networks
**EEUCH**	Energy-efficient unmanned aerial vehicle (UAV) assisted clustering
	hierarchical
**FoI**	Field of interest
**FSD**	Number of rounds spent when first SN dies
**IEE-LEACH**	Improved energy-efficient LEACH
**IoT**	Internet of Things
**LEACH**	Low energy adoptive clustering hierarchy
**LEACH-C**	LEACH centralized
**LoS**	Line of sight
**MR**	Main radio
**MST**	Mode switching time
**NB**	Current number of backoffs
**PEGASIS**	Power-efficient gathering in sensor information systems
**RR**	Rumor routing
**RTS**	Request to send
**SEP**	Stable energy protocol
**SNs**	Sensor nodes
**TEEN**	Threshold sensitive energy effective sensor network
**TDMA**	Time division multiple access
**UAV**	Unmanned aerial vehicle
**WRP**	Wireless routing protocol
**WSN**	Wireless sensor network
**WuC**	Wake-up call
**WuR**	Wake-up radio
**WuRx**	Wake-up receiver
